# Computational fluid dynamics as supporting technology for coronary artery disease diagnosis and treatment: an international survey

**DOI:** 10.3389/fcvm.2023.1216796

**Published:** 2023-08-31

**Authors:** Claudio Chiastra, Marco Zuin, Gianluca Rigatelli, Fabrizio D’Ascenzo, Gaetano Maria De Ferrari, Carlos Collet, Yiannis S. Chatzizisis, Diego Gallo, Umberto Morbiducci

**Affiliations:** ^1^ PoliTo^BIO^Med Lab, Department of Mechanical and Aerospace Engineering, Politecnico di Torino, Turin, Italy; ^2^ Department of Translational Medicine, University of Ferrara, Ferrara, Italy; ^3^ Interventional Cardiology Unit, Department of Cardiology, Madre Teresa Hospital, Padova, Italy; ^4^ Division of Cardiology, Department of Medical Sciences, Città Della Salute e Della Scienza Hospital, Turin, Italy; ^5^ Cardiovascular Center of Aalst, Aalst, Belgium; ^6^ Division of Cardiovascular Medicine, Miller School of Medicine, University of Miami, Miami, FL, United States

**Keywords:** coronary artery disease, technology, precision medicine, modeling and simulation, computational fluid dynamics, hemodynamics, wall shear stress, helical flow

## Abstract

**Background:**

Computational fluid dynamics (CFD) is emerging as an effective technology able to improve procedural outcomes and enhance clinical decision-making in patients with coronary artery disease (CAD). The present study aims to assess the state of knowledge, use and clinical acceptability of CFD in the diagnosis and treatment of CAD.

**Methods:**

We realized a 20-questions international, anonymous, cross-sectional survey to cardiologists to test their knowledge and confidence on CFD as a technology applied to patients suffering from CAD. Responses were recorded between May 18, 2022, and June 12, 2022.

**Results:**

A total of 466 interventional cardiologists (mean age 48.4 ± 8.3 years, males 362), from 42 different countries completed the survey, for a response rate of 45.9%. Of these, 66.6% declared to be familiar with the term CFD, especially for optimization of existing interventional techniques (16.1%) and assessment of hemodynamic quantities related with CAD (13.7%). About 30% of respondents correctly answered to the questions exploring their knowledge on the pathophysiological role of some CFD-derived quantities such as wall shear stress and helical flow in coronary arteries. Among respondents, 85.9% would consider patient-specific CFD-based analysis in daily interventional practice while 94.2% declared to be interested in receiving a brief foundation course on the basic CFD principles. Finally, 87.7% of respondents declared to be interested in a cath-lab software able to conduct affordable CFD-based analyses at the point-of-care.

**Conclusions:**

Interventional cardiologists reported to be profoundly interested in adopting CFD simulations as a technology supporting decision making in the treatment of CAD in daily practice.

## 1. Introduction

Coronary artery disease (CAD) continues to be the leading cause of morbidity and mortality in Western countries (1). From an epidemiological perspective, CAD affects approximately 200 million individuals and it is responsible for nearly 9 million deaths worldwide (2). CAD can be diagnosed using non-invasive or invasive tests, and the treatment options include pharmacological and interventional options. However, coronary angiography is still considered the gold standard technique for assessing CAD and providing guidance for percutaneous coronary interventions (PCI) (3). Thus, the development of novel diagnostic and therapeutic technologies has received considerable interest.

In recent years, computational fluid dynamics (CFD) has emerged as a methodology ready to shift to technology for cardiovascular medicine, potentially enhancing the understanding of pathophysiology, by simulating interventional procedures, and improving decision making regarding CAD treatment (4–8). CFD utilizes numerical techniques to solve and analyze problems involving fluid flows, including blood flow (4). Within the context of cardiovascular medicine, CFD has been extensively employed to investigate the role of hemodynamics in CAD pathophysiology (5, 9, 10). It has found widespread use in the functional assessment of CAD and in the elucidation of the role of hemodynamics in CAD initiation, progression, susceptibility and phenotype (4, 11, 12). Additionally, CFD has demonstrated its capability to describe and predict the hemodynamic responses before (13, 14) and after cardiovascular interventional procedures (15, 16), making it a valuable tool in the development and enhancement of intervention devices and treatment strategies (6, 17–21). Furthermore, current literature has highlighted that basic modeling concepts are now ripe for incorporation into the knowledge base of interventional cardiologists. In this regard, recent evidences have demonstrated the feasibility of independent management by interventional cardiologists of CFD-based technology for CAD-related predictions (22).

Until now, the adoption of CFD as supporting technology and the opportunities opened by its clinical application in the context of CAD have been hampered by the demanding computational cost to run simulations, especially when compared to current diagnostic imaging acquisitions. This has prevented the use of computational hemodynamics in large clinical studies, which in turn would be required to prove the utility of computer-based hemodynamic modelling, setting up a vicious cycle. New technological approaches are under development, either supplementing or replacing conventional CFD, with the goal of making cardiovascular modelling tasks compatible with clinical needs with respect to simulation runtimes (23). Despite these advancements, the input data required for the hemodynamic analysis are usually affected by uncertainties and measurement noise impacting the accuracy of the simulation results. Currently, there is a growing recognition on the need for appropriate methods of uncertainty quantification able to estimate the propagation of uncertainty from the model inputs to the simulation results, to increase their reliability and enable greater clinical impact (24, 25). Moreover, CFD is still perceived by interventional cardiologists as a technology for which most of them have never been trained. This represents a major barrier to its widespread adoption. For these reasons, the aim of this work was to conduct an independent, international survey to evaluate the awareness, knowledge, and interest of interventional cardiologists in utilizing CFD simulations for the management of CAD.

## 2. Methods

### 2.1. Study design

We designed a 20-item, international, anonymous survey to assess the awareness, knowledge, interest and use of CFD in the management of CAD. For this purpose, an electronic questionnaire in English language designed using the virtual Google Form, consisting of 20 multiple-choice (single or multiple answers) or open-ended questions, was sent via e-mail to suitable respondents identified by the authors. Specifically, all the authors were involved in spreading the survey sending via e-mail the virtual form to their contacts and potential participants, registering the number of invitations sent. All potential respondents were physicians with a cardiology certification, working as interventional cardiologists and directly involved in the diagnosis and treatment of CAD during their daily clinical practice. To evaluate the knowledge and utilization of CFD in a real-world context, no additional strict criteria (e.g., based on experience levels or the number of interventional procedures performed annually) were applied in the selection of respondents. Responses were recorded between May 18^th^, 2022, and June 12^th^, 2022, and the participation was voluntary. A reminder e-mail was dispatched to the recipients two weeks after the initial invitation to encourage their participation. Data security and the protection of participants’ data was guaranteed by the security measures applied by the Google Form platform, by ensuring access to the data only to the form creators and by not collecting identifiable personal information. Access to the data was restricted to the form creators only. Considering that no patient-specific, pre-procedural and post-procedural data were collected, and all participation by study sites was voluntary and anonymous, no ethics committee review was required.

### 2.2. Data collection

**Figure 1:**
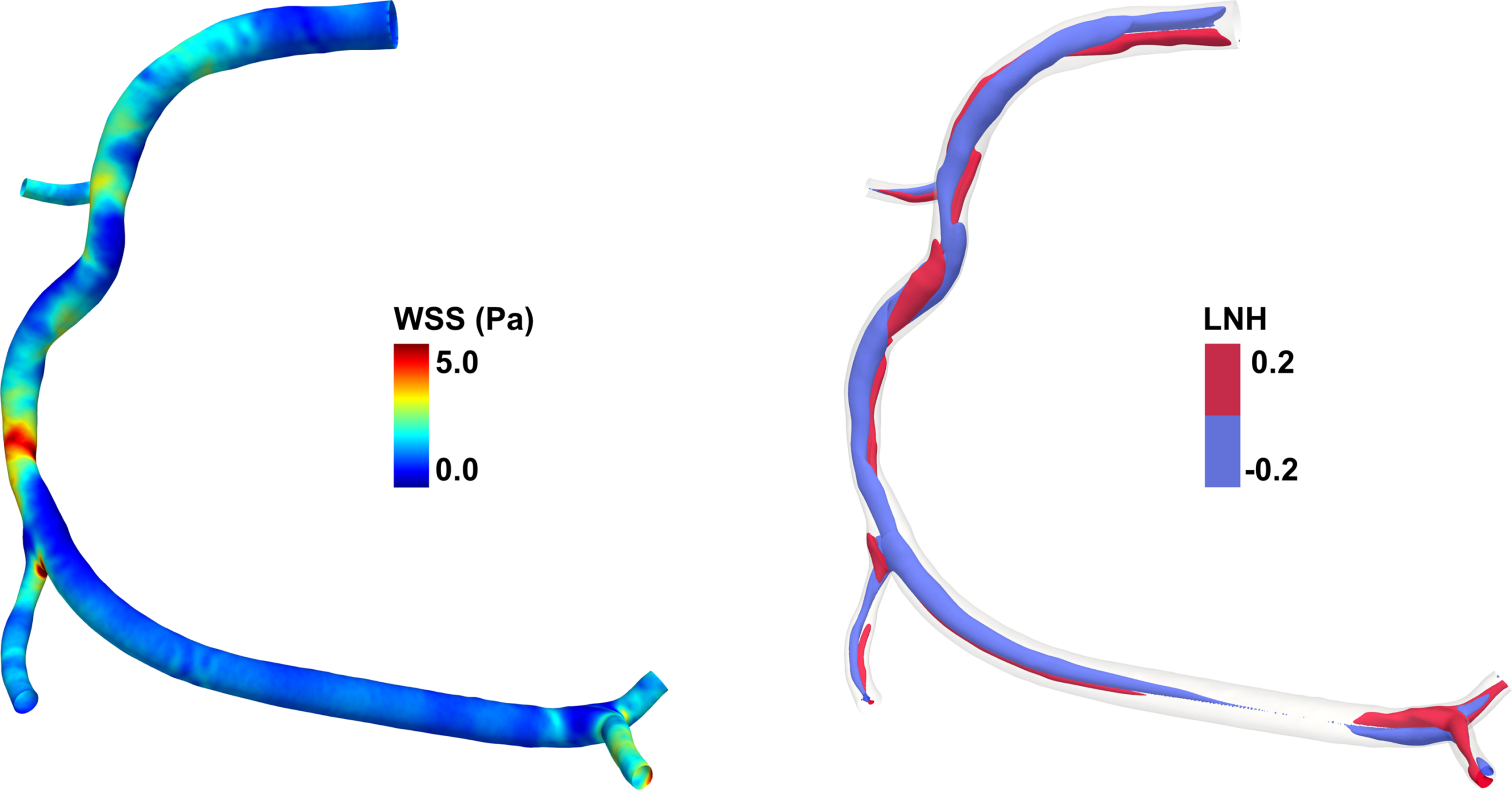
Example of hemodynamic quantities obtainable from computational fluid dynamics (CFD) simulations for a diseased right coronary artery model. (left) Color maps of wall shear stress (WSS) along the endothelial surface. The WSS is defined as the tangential stress due to the friction of the flowing blood on the endothelial surface. In the coronary artery model here reported, high WSS values are present at the stenosis region. (right) Isosurfaces of local normalized helicity (LNH) representing the counter-rotating helical flow structures that develop in the intravascular region of the coronary artery model. Positive/negative LNH values indicate right-handed/left-handed rotating fluid structures along the main flow direction and are displayed in red/blue, respectively. The diseased right coronary artery belongs to a patient recruited within the RELATE clinical trial (ClinicalTrials.gov Identifier: NCT04048005).

The survey was composed by three different sections: (i) respondents’ demographic details, such as age, sex, place of work and number of coronary interventional procedures performed yearly; (ii) questions regarding basic CFD knowledge, its potential application in CAD patients, and the role that hemodynamic quantities derived from CFD simulations, such as wall shear stress (WSS) and helical flow (HF), have in CAD (5, 26–29) (Figure 1 shows an explanatory example of the hemodynamic quantities obtainable from CFD simulations); (iii) questions exploring the interest of interventional cardiologists to obtain a simple orientation course (a) on the basic pathophysiological mechanisms related to local hemodynamics and (b) on the clinical implications of patient-specific CFD-based analysis in CAD, as well as (c) on the availability of software applications performing a reliable CFD-based analysis that can be used directly in the operating room by interventional cardiologists irrespective to the presence of specialized personnel (e.g., biomedical engineers). The full survey is available in the Supplementary Data.

### 2.3. Statistical analysis

A descriptive statistical analysis was performed to assess respondents’ general characteristics. Continuous variables were expressed as mean ± standard deviation (SD), while the non-normally distributed variables were presented in terms of median and confidence interval [CI]. Categorical variables were presented as proportions. The normally distributed continuous variables were compared using the student's *t*-test or ANOVA (with Bonferroni's *post hoc*), whereas the non-Normally distributed variables were compared using the Mann–Whitney *U*-test. Comparisons between categorical variables were performed using *χ*^2^-test or Fisher's exact test. A multivariate regression analysis was computed to identify independent associations with knowledge of CFD in CAD patients. Variables with *p* < 0.1 characterizing the univariate analysis were included in the multivariate model. A cut-off of 40 years was chosen to differentiate between “expert” interventional cardiologists (i.e., cardiologists with ostensibly >10 years of clinical practice) and those with less experience. The knowledge of the pathophysiological role of WSS and HF was defined as the correct answer to all the related questions in the survey. Statistical analyses were performed using SPSS package version 20.0 (SPSS, Chicago, IL, USA).

## 3. Results

### 3.1. Respondents’ characteristics

**Figure 2:**
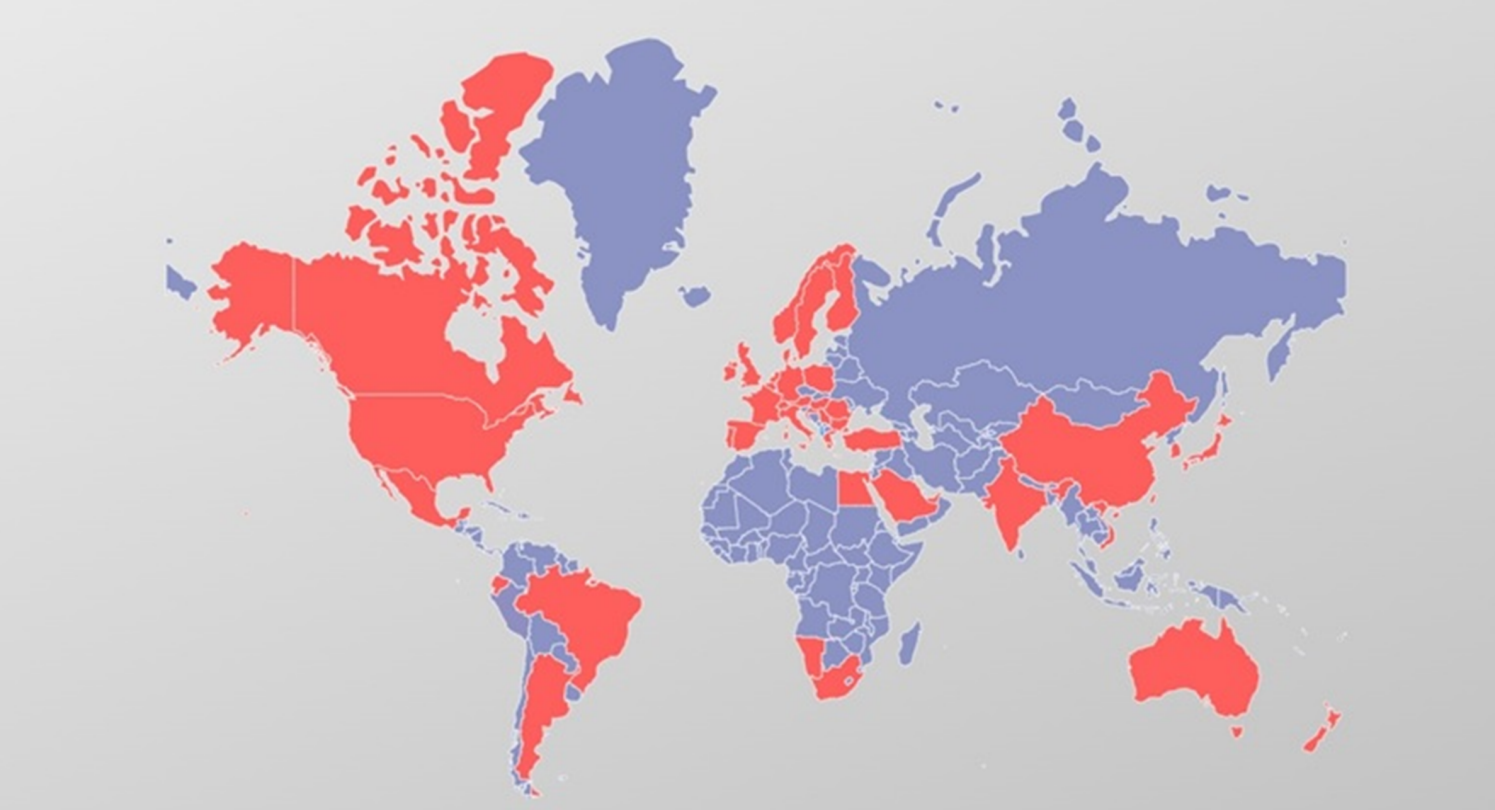
Geographical distribution of respondents. Countries of respondents are colored in red.

A total of 466 interventional cardiologists (mean age 48.4 ± 8.3 years, males 362) from 42 different countries (Figure 2) completed the survey out of 1015 invitations, resulting in a response rate of 45.9%. The demographic and general characteristics of the respondents are presented in Table 1. Most interventional cardiologists were males, based in Europe (59.2%) and America (23.3%) (Figure 2) and practice in non-university teaching hospitals (41.0%). More than half of respondents (64.5%) performed more than 100 PCI yearly. Male respondents were older than women (49.0 ± 8.7 vs. 45.4 ± 6.0 years, *p* < 0.0001) and with a greater interventional experience (16.9 ± 8.7 vs. 12.0 ± 6.4 years, *p* < 0.0001). A further sub-analysis, stratifying respondents by continents, is provided into Supplementary Table S1.

**Table 1:** General characteristics of respondents.

	*N* = 446
Mean age (years) [min-max]	48.4 ± 8.3 [29–71]
Males, *n* (%)	362 (81.2)
Years in practice (years) [min-max]	15.9 ± 8.5 [1–39]
Continent of respondents, *n* (%)	
Europe	264 (59.2)
America	104 (23.3)
Asia	62 (13.9)
Oceania	10 (2.2)
Africa	6 (1.3)
Type of hospital, *n* (%)	
University hospital	131 (29.4)
Non-university—teaching hospital	183 (41.0)
Non-university—non-teaching hospital	131 (29.4)
Other	1 (0.2)
Number of coronary interventional procedures (per year), *n* (%)	
<25	5 (1.1)
26–50	3 (0.7)
51–75	17 (3.8)
76–99	48 (10.8)
100–125	85 (19.1)
126–150	91 (20.4)
151–175	94 (21.1)
176–199	49 (11.0)
>200	54 (12.1)

### 3.2. How familiar cardiologists are with CFD

Among all the respondents, 66.6% (*n* = 297) declared to be familiar with the term CFD; of these, 65.2% declared to be aware of clinical applications of CFD in CAD and coronary artery interventions. A further sub-analysis evidenced that most respondents declaring to have knowledge on CFD applications were younger (45.7 ± 7.2 vs. 53.6 ± 8.0 years old, *p* < 0.0001) and with less years of practice (13.4 ± 7.4 vs. 21.4 ± 8.3 years, *p* < 0.0001) compared to those who were unaware of CFD uses in CAD management. Most of respondents declared to be aware of CFD-based analysis applied to optimize existing interventional techniques (22.5%), followed by those deeming CFD simulations necessary to evaluate local hemodynamic quantities related with CAD (19.0%) or to quantify not invasively the fractional flow reserve (FFR) (18.7%) (Table 2), an index used for the assessment of the functional severity of coronary stenoses.

**Table 2:** Applications of CFD analysis known by respondents.

Known application of CFD	*N* = 320
Quantification and analysis of CFD-derived (non-invasive) fractional flow reserve	60 (18.8)
Quantification and analysis of wall shear stress (WSS) as a risk factor for coronary artery disease	56 (17.5)
Optimization of existing interventional techniques	72 (22.5)
Evaluation of new devices, such as stents and balloons (assessment of restenosis or thrombosis risk)	36 (11.2)
Evaluation of hemodynamic indices related with CAD	61 (19.0)
Prediction of MACE prior PCI	7 (2.1)
Prediction of MACE after PCI	28 (8.7)

CAD, coronary artery disease; MACE, major adverse cardiovascular events; PCI, percutaneous coronary intervention; WSS, wall shear stress.

### 3.3. How familiar cardiologists are with coronary wall shear stress and helical flow

Only 118 respondents (26.5%) correctly answered to the question exploring the knowledge of the role of WSS in the atherogenic process. Conversely, 153 interventional cardiologists (34.3%) identified the correct answer regarding the pathophysiologic role of WSS after coronary artery stenting. Finally, 48.2% (*n* = 215) respondents correctly attributed an atheroprotective role to HF in the coronary arteries. Notably, in all the three previous questions no significant differences were registered in age between those who answered correctly or not.

### 3.4. Interest on CFD simulations for daily interventional practice

**Figure 3:**
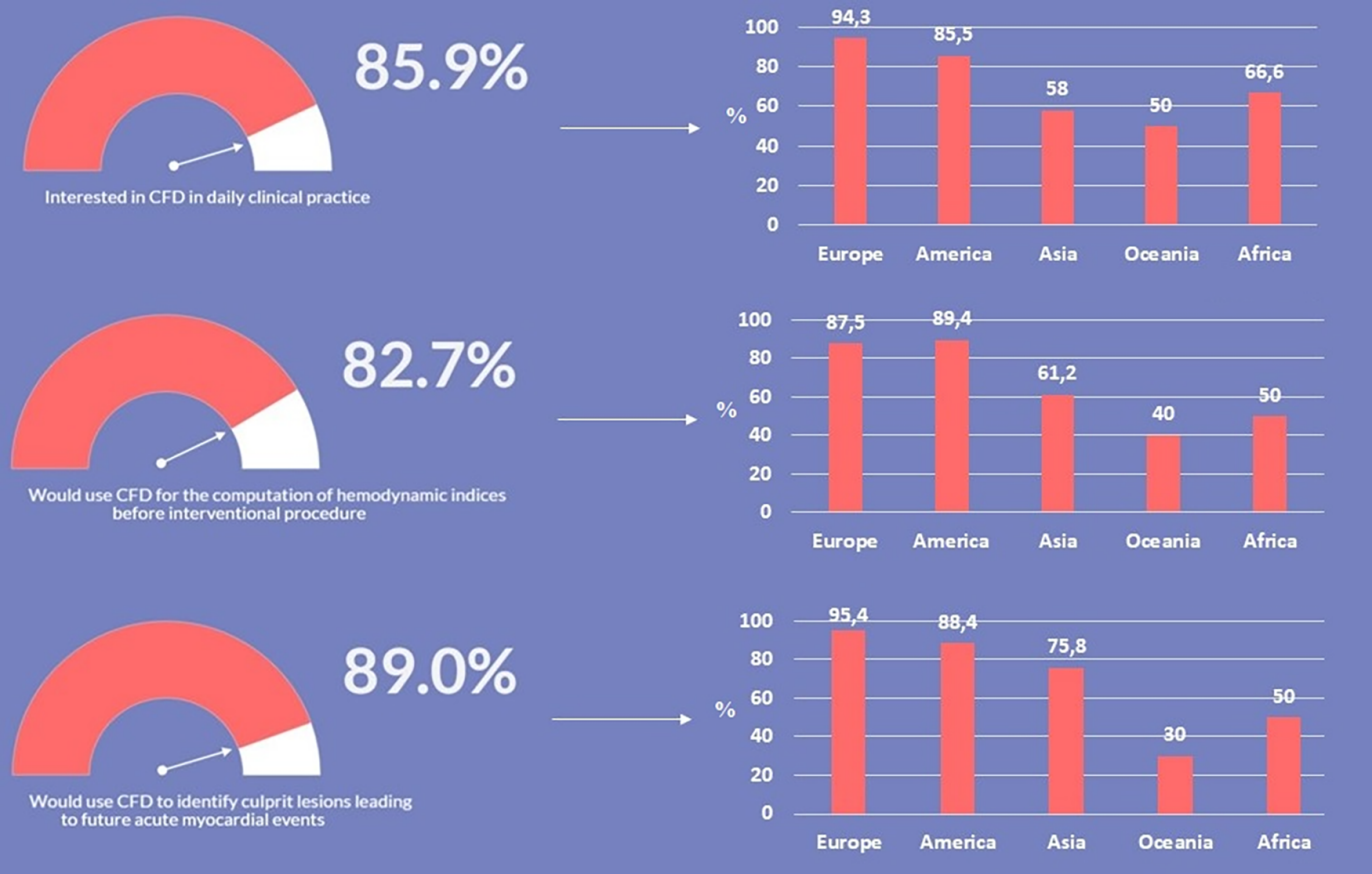
Interest in the use and application of computational fluid dynamics (CFD) in coronary artery disease.

**Figure 4:**
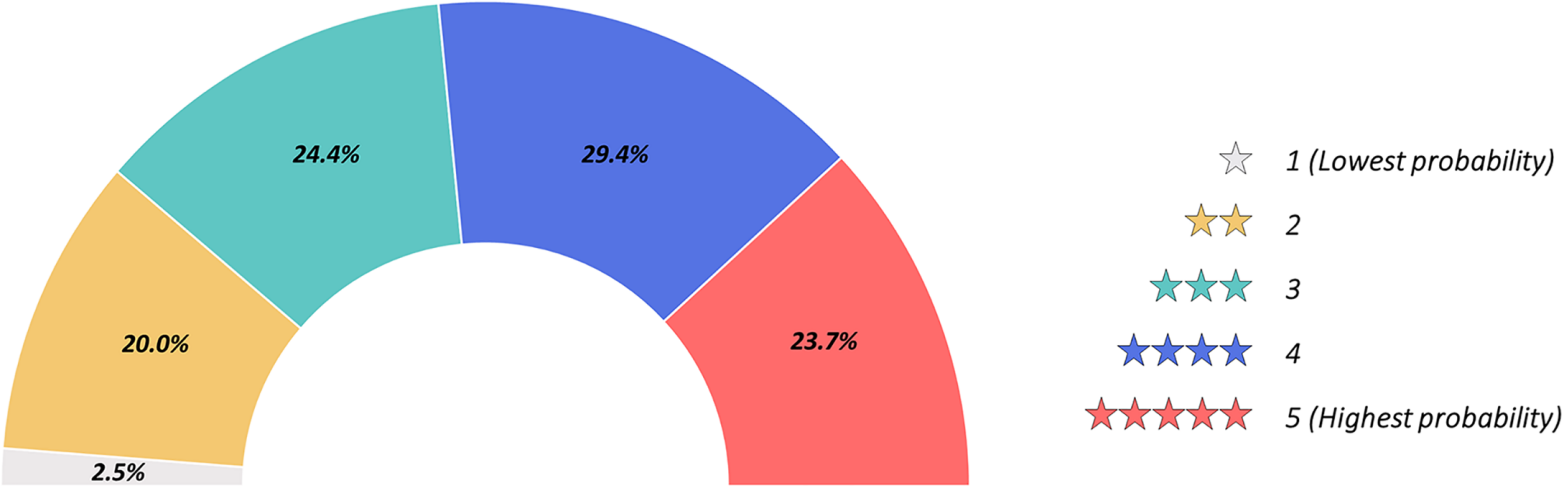
Probability for computational fluid dynamics (CFD) use in future daily interventional practice graded from one to five. 1, lowest probability; 5, highest probability.

**Figure 5:**
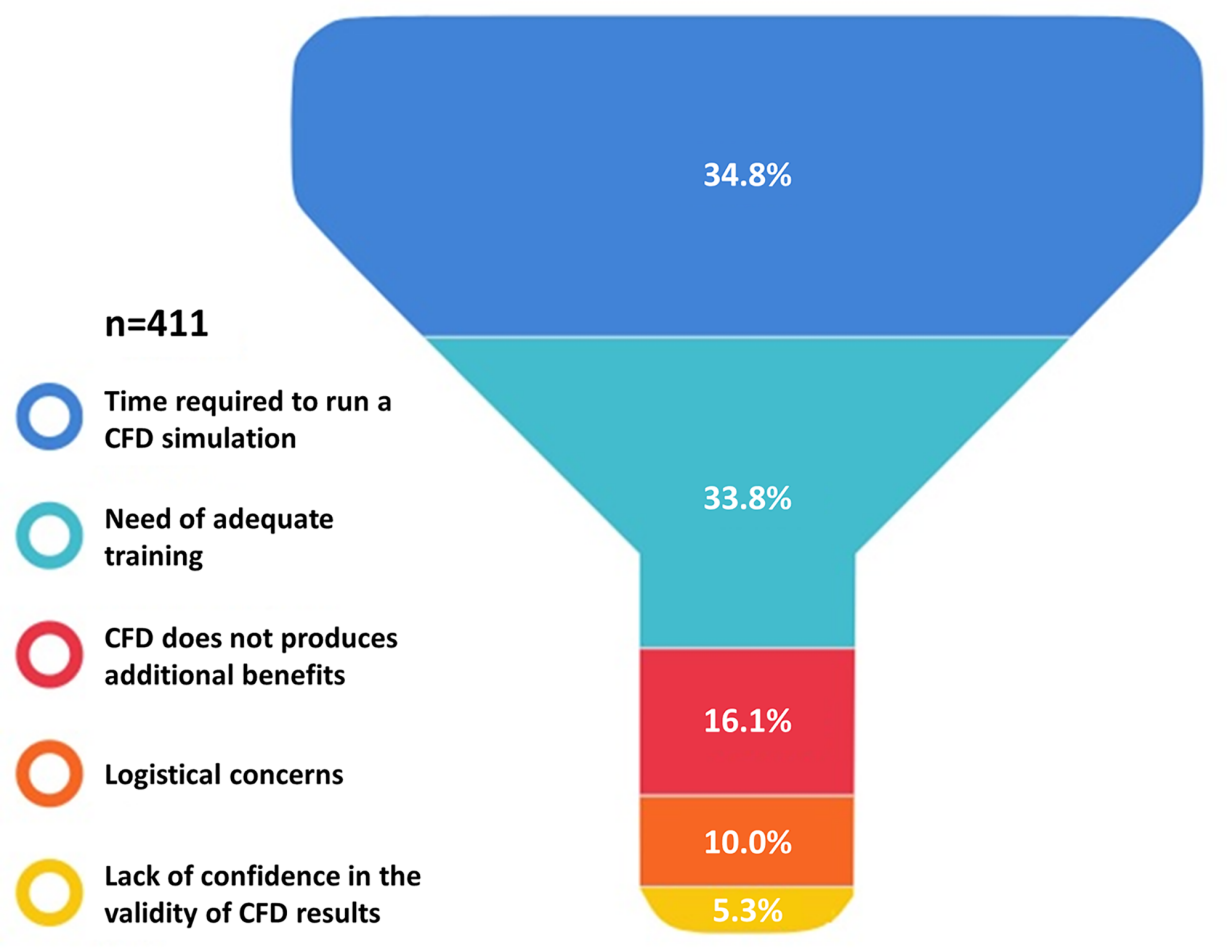
Reservations about the applications of computational fluid dynamics (CFD) in the clinical practice.

As presented in Figure 3, irrespectively of age and years of practice the 85.9% (*n* = 383) of respondents would consider CFD-based analysis in daily interventional practice, for the evaluation of WSS and HF before procedures (82.7%, *n* = 369) and for the application of a baseline CFD simulation (89.0%, *n* = 397) to preliminary identify culprit lesions potentially leading to future acute myocardial infarctions. Regarding the interest in introducing CFD analysis in daily clinical practice, on a scale from one to five, where one represents the lowest and five the highest probability, 29.4% and 23.7% of respondents gave a score of four and five, respectively (Figure 4). The most common concerns related to the use of CFD in the clinical practice are the amount of time required to run a simulation and the need for proper training (34.8% and 33.8%, respectively) (Figure 5).

### 3.5. Interest to obtain adequate training and appropriate software

**Figure 6:**
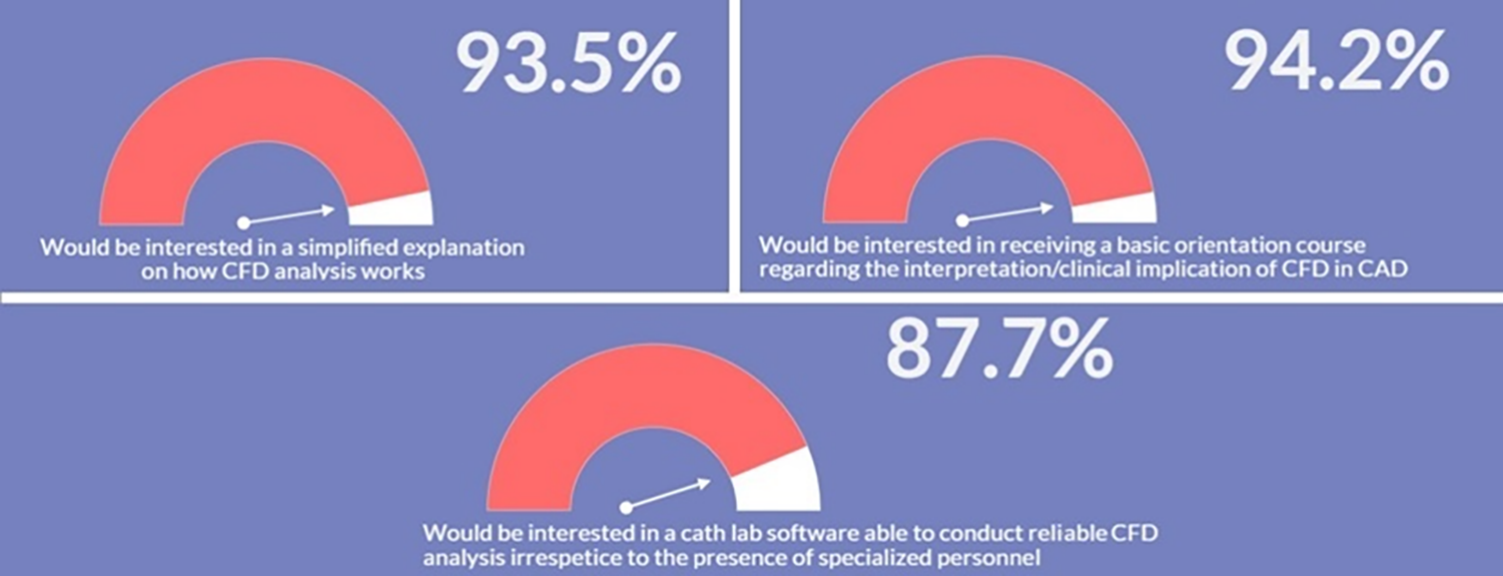
Interest in receiving an adequate training and modern software for computational fluid dynamics (CFD).

Most respondents (93.5%, *n* = 417) were interested in obtaining simple and basic explanation regarding CFD simulations and their application in CAD (Figure 6). Similarly, the majority of those surveyed declared to be interested in: (i) receiving a brief foundation course on the basic pathophysiological mechanisms related to local hemodynamics and on the clinical implications of CFD analysis in CAD (non-invasive functional assessment to detect flow-limiting stenosis for CAD treatment, prediction of adverse events prior or after intervention) (94.2%, *n* = 420); (ii) the availability of a software performing reliable patient-specific CFD simulations at the point-of-care irrespective to the presence of specialized personnel such as biomedical engineers (87.7%, *n* = 391) (Figure 6). Multivariate regression analysis evidenced that age <40 years (OR: 1.13, 95% CI: 1.08–1.19, *p* < 0.0001), working in a university hospital (OR: 1.85, 95% CI: 1.39–2.26, *p* < 0.001) and knowledge of the pathophysiological role of WSS and HF (OR: 1.12, 95% CI: 1.08–1.16, *p* = 0.01) were independently associated to consideration of CFD simulations in future daily clinical practice (Table 3).

**Table 3:** Multivariate regression analysis identifying the predictors of future use of CFD analysis in daily practice.

	Univariate			Multivariate		
	OR	95% CI	*p*	OR	95% CI	*p*
Age <40 years	1.18	1.11–1.25	<0.001	1.13	1.08–1.19	<0.0001
Gender (male vs. female)	0.73	0.34–1.36	0.38	–		
Continent (Europe as reference)	1.13	0.93–1.08	0.42	–		
University hospital (non-university-non-teaching hospital as reference)	1.98	1.46–2.21	<0.001	1.85	1.39–2.26	<0.001
≥100 CAD procedures per year (<100 as reference)	1.39	0.89–1.24	0.62	–		
Knowledge of pathophysiological role of WSS and HF (yes vs. no)	1.24	1.13–1.37	0.001	1.12	1.08–1.16	0.01

## 4. Discussion

The findings of this survey suggest that most interventional cardiologists are interested in computer simulations of coronary hemodynamics and in their applications to CAD. In fact, 85.9% of respondents would consider CFD in daily interventional practice, while 89.0% would be interested in using computational simulations to pre-operatively identify the culprit lesions that may lead to future major cardiovascular events (MACE), typically including acute myocardial infarction, stroke and cardiovascular mortality (30). Moreover, 94.2% of participants are interested in receiving a brief and basic orientation course regarding the pathophysiological mechanisms associated with CFD analysis in CAD.

Recently, several surveys have been conducted in the field of interventional cardiology on various topics, such as the impact and management of PCI complications (31), the implementation of FFR in daily practice (32), and the adoption of American College of Cardiology appropriate use criteria for revascularization (33), among other areas of interest. However, our investigation stands out as the first attempt to assess and shed light on the current knowledge and interest regarding the use of CFD as a supporting technology in CAD management and decision making. The number of respondents align with or, in certain cases (32), exceed that of previous surveys. Moreover, the response rate was comparable (32) or higher (31, 33) than that reported by previous studies.

Examining the results of our survey in detail, it become apparent that despite 66.6% of respondents expressed familiarity with the term “CFD”, there was a widespread self-awareness of a skill gap in dealing with simulations as technology users and interpreting CFD-derived hemodynamic quantities such as WSS and HF. These results reflect a lack of basic training and education in computational medicine and its specific applications to CAD. Indeed, the integration of CFD with clinical imaging candidates to be the technological solution for minimally invasive patient assessment and for the obtainment of patient-specific evaluations of the hemodynamic risk associated with CAD, thus promoting the diffusion of precision medicine practices among interventional cardiologists (34–36).

As highlighted by our results, younger cardiologists exhibit greater level of confidence on CFD simulations. This suggests that CFD may become a routine aspect of daily practice in the near future, particularly for predicting physiological hemodynamic responses following cardiovascular interventional procedures (Table 4) (5, 9–11, 16–19, 22, 26, 27, 37–65). The reasons are probably multifactorial. Firstly, young generations tend to be more comfortable with and open to adopting new technologies, in particular computer models. Secondly, while young interventionalists are being trained, CFD techniques are being widely utilized for research purposes, despite their limited use in clinical practice (5). However, when we tested respondents’ knowledge of pathophysiological concepts related to or emerging from CFD-based applications to CAD, only a minority identified the correct answers. This indicates that the theoretical background for both the application of CFD simulations and the interpretation of their output in the clinical framework is still lacking. Respondents were often unfamiliar with the role of WSS in promoting (i) atherosclerosis at the early stage (29, 48), (ii) its progression (29, 48), or (iii) its impact on neointimal proliferation after stenting (66–68). Similarly, 51.8% of respondents answered incorrectly regarding the atheroprotective role of HF in coronary arteries (26–28). It seems that some respondents are updated regarding the significance of the hemodynamic quantities proposed by experts in CFD for cardiovascular flows, but they are not fully aware of the underlying links between specific hemodynamic patterns and biological aggravating events at the arterial wall.

**Table 4:** Computational fluid dynamics (CFD) applications in diagnosis and treatment of coronary artery disease (CAD).

Application	Clinical context of use	Currently used in the clinical practice (Y/N)	Current critical issues	Selected references
Virtual FFR (CFD-derived non-invasive FFR)	Non-invasive functional assessment to detect flow-limiting stenosis for CAD treatment	Y	• Imaging modality for vessel reconstruction (radiation dose, resolution) • Challenges in the reconstruction of coronary vessels • Challenges in the definition of patient-specific boundary conditions	(11, 43–45)
WSS analysis	Evaluation of the onset and progression of atherosclerotic plaques	N	• Imaging modality for vessel reconstruction (radiation dose, resolution) • Accurate 3D reconstruction of coronary vessels • Definition of patient-specific boundary conditions • Computational costs	(5, 9, 10, 46–49)
	Prediction of MACE prior PCI			(5, 22, 50–53)
Intravascular flow analysis	Evaluation of the impact of intravascular flow features (e.g., helicity) on atherosclerotic plaque development	N	• Imaging modality for vessel reconstruction (radiation dose, resolution) • Accurate 3D reconstruction of coronary vessels • Definition of patient-specific boundary conditions • Computational costs	(26, 27, 54)
Virtual stenting	Treatment planning through the evaluation of stent-induced flow disturbances to evaluate ISR and ST risk and to predict MACE after PCI	N	• Imaging modality for stented vessel reconstruction (radiation dose, resolution) • Accurate 3D reconstruction of stented coronary vessels • Definition of patient-specific boundary conditions • Computational costs	(16–19, 55–58, 65)
	Optimization of existing interventional techniques or development of new ones	N	• Computational costs	
	Optimization of existing devices or development of new ones	N (used by stent manufacturers)	• Computational costs	
Mass transport analysis	Prediction of LDL concentration polarization profiles at the luminal surface as predictor of CAD	N	• Imaging modality for vessel reconstruction (radiation dose, resolution) • Accurate 3D reconstruction of stented coronary vessels • Definition of patient-specific boundary conditions and arterial wall transport properties • Computational costs	(59, 60–64)
	Profiling of drug concentration in coronary arteries treated with drug eluting stents to optimize drug release	N		(37–42)

FFR, fractional flow reserve; WSS, wall shear stress; MACE, major adverse cardiovascular events; PCI, percutaneous coronary intervention; ISR, in-stent restenosis; ST, stent thrombosis; LDL, low-density lipoproteins.

CFD is a promising technology with the potential to provide researchers and clinicians with a deeper understanding of blood flow patterns, pressure gradients, and other hemodynamic variables for investigating the mechanisms underlying CAD. Furthermore, CFD can be utilized for device design and optimization, offering opportunities to enhance medical interventions and management of CAD. Additionally, patient-specific CFD can be employed in the context of precision medicine, e.g., to aid in preoperative planning and risk assessment, and for prognostic purposes, thus empowering interventional cardiologists to make informed decisions and improve patient outcomes during CAD-related procedures. Despite its potential, the widespread adoption of CFD in cardiology clinical practice is characterized by several challenges. These challenges encompass the necessity for additional validation studies and standardization, logistical and computational resource availability, seamless integration with existing clinical workflows, accessibility of CFD software and expertise, efficient data accessibility and sharing, and regulatory considerations. Recent technological advancements are addressing these challenges. The availability of increasingly powerful computational resources at lower costs along with the development of dedicated and user-friendly software for calculating hemodynamics quantities of interest for cardiologists [e.g., virtual FFR and WSS descriptors (4)] is making CFD simulations more accessible. Furthermore, the ability to run CFD simulations in the cloud holds the potential to eliminate the need for workstations directly in the cath-lab, thereby reducing logistic and computational resource barriers. The integration of CFD with existing diagnostic imaging techniques, such as coronary angiography, is already feasible. Recent clinical studies have successfully demonstrated the computation of WSS descriptors with timeframes compatible with clinical practice based on CFD simulations performed by cardiologists (22, 69, 70).

Along with the diffusion and utilization of CFD in CAD, the survey emphasized the critical need to expand educational opportunities for interventional cardiologists to enhance their knowledge of this technique. In fact, the survey responses indicate that these potential educational efforts would be well received. Our multivariate regression analysis showed that interventional cardiologists aged less than 45 years old, working in a university hospital and having adequate knowledge of CFD-based hemodynamic characterization were more likely to adopt CFD in future interventional practice. These results imply the possibility of CFD remaining a technique limited to academic environments among young interventionalists. Therefore, it would be essential to disseminate fluid mechanics and CFD basic knowledge to the future generations of interventional cardiologists by developing or integrating education on basic fluid mechanics and CFD into fellowship courses and specialized sessions of interventional congresses, thereby facilitating the expansion of their didactic curriculum. This educational approach will pave the way to even more patient-tailored strategies with the potential of improving patient outcomes. This calls for the development of innovative and collaborative programs in leading centers with the establishment of dedicated CFD teams involving trained interventional cardiologists, cardiac surgeons, and biomedical engineers. In this regard, nearly 90% of respondents expressed their belief that having dedicated and user-friendly software capable of autonomously conducting reliable CFD simulations, independent of dedicated personnel, would be beneficial in everyday clinical practice. This tendency reinforces the necessity of a streamlined and user-friendly methodology that simplifies cardiovascular hemodynamic calculation, requiring less time and lower costs while still ensuring model accuracy.

The main strength of this study is represented by the higher response rate obtained from 42 different countries located in five continents. Overall, the results of the survey may realistically reflect the attitude and knowledge of interventional cardiologists on the use of CFD simulations in CAD worldwide. Nevertheless, this study faces some limitations. Firstly, the selection of respondents was based on the diffusion of the survey through the worldwide contacts of the authors. However, the number of participants partially mitigate this aspect. Secondly, demographic and professional data of nonrespondents were not available for comparison with respondents. Therefore, we hypothesize that interventional cardiologists who completed the survey may have been more confident or interested in the subject, thus potentially biasing the results toward overestimating exposure and training in CFD analysis. However, it is important to note that our study participants from the USA exhibited demographic characteristics comparable to the total active population of interventional cardiologists in USA, according to the recent report by the Association of American Medical Colleges (71). This includes a substantial majority of male cardiologists (91.8%) and a higher proportion of younger practitioners (83.9% under age 55). While detailed demographic data were unavailable for other continents, the characteristics of respondents are consistent with the data from the USA, providing additional support for the validity of our study concerning the surveyed population. Lastly, a potential limitation lies in the possibility of bias introduced by the formulation of some questions. In particular, the wording and structure of the questions may have influenced the way participants interpreted and responded to them. Nevertheless, we emphasize that if such a bias is introduced, it is not significant enough to alter the overall findings of the study, which indicate a general interest among interventional cardiologists in CFD and a lack of training in CFD simulations and interpretation of CFD results.

## 5. Conclusions

In this international survey investigating the understanding and utilization of CFD as supporting technology for CAD diagnosis and treatment, the majority of interviewed interventional cardiologists expressed interest in the use of computer simulations of coronary hemodynamics and in their potential applications to CAD in everyday clinical practice. The survey revealed a prevalent self-perceived deficiency in using simulations and interpreting CFD-derived hemodynamic quantities such as WSS and HF. This observation underscores the significance of addressing the lack of basic training and education in computational medicine for CAD. It is essential to prioritize the enhancement of educational opportunities in CFD applications, particularly for young interventional cardiologists, as well as establish dedicated CFD teams consisting of trained interventional cardiologists, cardiac surgeons, and biomedical engineers. Additionally, further research is necessary to develop user-friendly CFD software. All of these measures are crucial for promoting the widespread adoption of CFD in clinical practice.

## Data Availability

The raw data supporting the conclusions of this article will be made available by the authors upon request.
