# Supplementary material for: Computational fluid dynamics as supporting technology for coronary artery disease diagnosis and treatment: an international survey
Source: Front Cardiovasc Med. 2023 Aug 31;10:1216796. doi: 10.3389/fcvm.2023.1216796 (PMC10501454; doi:10.3389/fcvm.2023.1216796)
Supplement: Supplementary file 1 [file Datasheet1.pdf]

Supplementary Material

Computational Fluid Dynamics as Supporting Technology for Coronary Artery Disease Diagnosis and Treatment: An International Survey

Claudio Chiastra, Marco Zuin, Gianluca Rigatelli*, Fabrizio D'Ascenzo, Gaetano Maria De Ferrari, Carlos Collet, Yiannis S. Chatzizisis, Diego Gallo, Umberto Morbiducci*

*** Correspondence:** Gianluca Rigatelli: jackyheart@libero.it; Umberto Morbiducci: umberto.morbiducci@polito.it

1 Supplementary Data.

Survey

1. Your Gender

   ☐ Male

   ☐ Female

2. Your age:___(years)

3. Years in practice:____(years)

4. Your country:________

5. Type of hospital:
   ☐ University hospital
   ☐ Non-university, teaching hospital
   ☐ Non-university, non-teaching hospital
   ☐ Other

6. How many coronary interventions do you perform yearly?
   ☐ <25
   ☐ 26-50
   ☐ 51-75
   ☐ 76-99
   ☐ 100-125
   ☐ 126-150

☐ 151-175
☐ 176-199
☐ >200

7. Are you familiar with the term computational fluid dynamics "CFD" analysis?
   ☐ Yes
   ☐ No

8. If yes, are you aware of the utility and clinical applications of CFD in coronary artery disease and cardiovascular interventions?
   ☐ Yes
   ☐ No

9. If yes, with which of the following applications of CFD are you most familiar with? **(You can choose more than one answer).**
   ☐ Quantification and analysis of CFD-derived (non-invasive) fractional flow reserve
   ☐ Quantification and analysis of wall shear stress (WSS) as a risk factor for coronary artery disease
   ☐ Optimization of existing interventional techniques
   ☐ Evaluation of new devices, such as stents and balloons (assessment of restenosis or thombosis risk)
   ☐ Evaluation of hemodynamic indices related with coronary artery disease
   ☐ Prediction of major adverse cardiovascular events (MACE) prior to percutaneous coronary interventions (PCIs)
   ☐ Prediction of MACE after PCIs

10. Please select the answer that best describes wall shear stress (WSS)
   ☐ When high, WSS influences the initiation and/or progression of atherosclerosis in coronaries
   ☐ When low, WSS influences the initiation and/or progression of atherosclerosis in the coronaries
   ☐ WSS is involved in the processes leading to plaque progression, erosion and rupture
   ☐ WSS plays a role in the development of the atherosclerosis only in patients with previous coronary artery events
   ☐ WSS plays a role in the development of atherosclerosis only in patients with vascular comorbidities, such as arterial hypertension or peripheral artery disease
   ☐ WSS has no effect on the atherosclerotic process

11. Please select the correct answer: after coronary stenting, low wall shear stress (WSS)
   ☐ Promotes neointimal proliferation only at the stent edges
   ☐ Does not promote neointimal proliferation within the stent and at the stent edges
   ☐ Promotes neointimal proliferation only within the stent
   ☐ Promotes neointimal proliferation within the stent and at the stent edges
   ☐ Has no influence on the vascular remodelling of the vessel

12. Please select the correct statement regarding the presence of helical flow within the coronary artery
    ☐ Helical flow is atheroprotective in healthy subjects
    ☐ Helical flow promotes atherosclerosis
    ☐ Helical flow has no influence on the atherosclerotic process

13. Would you consider CFD analysis useful in your interventional practice?
    ☐ Yes
    ☐ No
    ☐ I do not know

14. If yes, would you consider CFD for the computation of hemodynamic indices, such as WSS or helical flow before an interventional procedure?
    ☐ Yes
    ☐ No
    ☐ I do not know

15. Considering that baseline CFD analysis could identify culprit lesions leading to future acute myocardial events, do you think that this technology would be useful in selected patients before an elective procedure?
    ☐ Yes
    ☐ No
    ☐ I do not know

16. On a scale from 1 to 5, with 5 representing the highest probability and 1 the lowest probability, how likely are you to introduce CFD analysis in coronary artery interventions in your daily clinical practice?
    ☐ 1
    ☐ 2
    ☐ 3
    ☐ 4
    ☐ 5

17. If you have reservations about the applications of CFD in the clinical practice, what are the reasons? Please mark the answers that best suit to you: **(You can choose more than one answer).**
    ☐ I am not sure whether CFD analysis provides additional benefits compared with the current standard of care
    ☐ I do not trust the validity of the CFD analysis results
    ☐ I have concerns about receiving adequate training in the field of CFD analysis
    ☐ I have reservations associated with the time needed to run CFD analyses
    ☐ I have reservations associated with the logistical aspects of the conduction of CFD analysis

18. Do you think that a simplified and straightforward explanation on how the CFD analysis works and on its applications in coronary artery disease would be helpful for your comprehension and use of CFD in the daily practice?
    - ☐ Yes
    - ☐ No
    - ☐ I do not know

19. Would you be interested in receiving a brief, basic orientation course in the interpretation of the basic pathophysiological mechanisms associated with the CFD analysis and clinical implications of the CFD analysis in coronary artery disease?
    - ☐ Yes
    - ☐ No
    - ☐ I do not know

20. Would you be interested in a cath-lab software able to conduct reliable CFD analysis at the point-of-care irrespective to the presence of specialized personnel (e.g. biomedical engineers)?
    - ☐ Yes
    - ☐ No
    - ☐ I do not know

2 Supplementary Tables

Supplementary Table 1. General characteristics of respondents, stratified by continents. *p<0.05 compared to mean age of other continents.

	**Europe N=264**	**America N=104**	**Asia N=62**	**Oceania N=10**	**Africa N=6**	**p (ANOVA)**
Mean age (years) [min-max]	47.2±8.7* [29-68]	50.9±8.2 [31-71]	48.4±6.0 [32-61]	50.1±9.0 [37-52]	49.0±2.2 [47-53]	0.005
Males, n (%)	208 (78.8)	82 (78.8)	59 (95.2)	8 (80.0)	5 (83.3)	0.52
Years in practice (years) [min-max]	15.3±8.8 [1-36]	17.0±8.9 [2-39]	16.4±4 [6-28]	17.2±9.2 [5-28]	17.6±4.3 [6-29]	0.48
Type of hospital, n (%)						
University hospital	96 (36.4)	28 (26.9)	5 (8.1)	2 (20.0)	0	
Non-university - teaching hospital	98 (37.1)	44 (42.3)	33 (53.2)	5 (50.0)	3 (50.0)	0.04
Non-university - non-Teaching hospital	69 (26.1)	32 (30.8)	24 (38.7)	3 (30.0)	3 (50.0)	0.74
Other	1 (0.4)	0	0	0	0	0.99
Number of coronary interventional procedures (per year), n (%)						
<25	4 (1.5)	0	1 (1.6)	0	0	0.98
26-50	3 (1.1)	0	0	0	0	0.99
51-75	7 (2.7)	5 (4.8)	4 (6.5)	1 (10.0)	0	0.03
76-99	26 (9.8)	14 (13.5)	7 (11.3)	0	0	0.59
100-125	42 (15.9)	28 (26.96)	14 (22.6)	0	1 (16.7)	0.05
126-150	51 (19.3)	19 (18.3)	14 (22.6)	4 (40.0)	3 (50.0)	0.007
151-175	58 (22.0)	20 (19.2)	12 (19.4)	3 (30.0)	1 (16.7)	0.67
176-199	31 (11.7)	16 (15.4)	2 (3.2)	0	0	0.04
>200	42 (15.9)	2 (1.9)	8 (12.9)	2 (20.0)	0	0.03
